# Enhanced cytotoxicity of a redox-sensitive hyaluronic acid-based nanomedicine toward different oncocytes via various internalization mechanisms

**DOI:** 10.1080/10717544.2019.1709919

**Published:** 2020-01-02

**Authors:** Yunai Du, Sheng Wang, Tianhao Zhang, Dongsheng He, Jiasheng Tu, Yan Shen

**Affiliations:** Center for Research Development and Evaluation of Pharmaceutical Excipients and Generic Drugs, Department of Pharmaceutics, School of Pharmacy, China Pharmaceutical University, Nanjing, China

**Keywords:** Hyaluronic acid, vitamin E succinate, internalization mechanism, anticarcinoma

## Abstract

Receptor-mediated active targeting and tumor microenvironment responsive systems from polymeric micelles have been studied for rapid cellular internalization and triggered drug release. Previously we have constructed redox-responsive polymeric micelles composed of vitamin E succinate conjugated hyaluronic acid (HA-ss-TOS), which are able to actively target CD44 proteins and quickly release loaded drugs upon exposure to high levels of glutathione (GSH) in tumor cells. In the present study, we found that despite different cellular internalization mechanisms, micelles showed strong antineoplastic effects on 4T1 and B16F10 cells due to redox responsiveness. HA-ss-TOS-PTX micelles exhibited an excellent tumor targeting ability and prolonged retention time compared to Taxol *in vivo*. In addition, a superior antitumor effect was achieved compared to PTX-loaded insensitive micelles (HA-TOS-PTX) and Taxol. Our results revealed that PTX-loaded HA-ss-TOS micelles could enhance the antineoplastic efficacy of PTX for breast cancer and melanoma treatment and, thus, deserve further attention.

## Introduction

Clinical studies have found that although nano-drugs can effectively reach the tumor site, limited amounts are transported into tumor cells, resulting in decreased anti-tumor efficacy (Stras et al., 2016). Most of the cytotoxic drugs currently used in the clinic are required to enter cancer cells to exert inhibitory functions (Bailly 2014; Dissanayake et al., 2017; Jin et al., 2019). Therefore, in order to kill tumor cells, it is inevitable to elevate the concentration of drugs inside tumor cells (Chen et al., 2016). It is well known that tumor cells usually overexpress specific receptors on their membranes, which are potential targets for select ligands, such as antibodies (Dumont et al., 2019; Liu et al., 2019), transferrin (Ke and Xiang 2018; Venkatesan et al., 2019), hyaluronic acid (Jeong et al., 2019; Wang et al., 2019), etc. By coupling these ligands onto the surface of nancarriers, their affinity toward tumor cells can be dramatically improved, thereby increasing the intratumor accumulation of chemotherapeutics via receptor-mediated internalization.

Bio-responsive nanocarrier delivery systems are dependent on the physiological characteristics of the tumor microenvironment for charge reversal, triggered drug release and other functions (Cheng et al., 2015; Chen et al., 2019). Biological response signals in the tumor microenvironment include extracellular and intracellular pH changes or specific enzymes, a strong reduction environment in the tumor cells and other signals (Tang et al., 2018; Uthaman et al., 2018). The glutathione (GSH) level in tumor cells is up to 2–20 mM, which is 4–10 times higher than that in normal cells and nearly 1000 times than that in the extracellular fluid and blood(Huang et al., 2018; Ling et al., 2019). Therefore, a relatively strong reduction environment in tumor cells could be a promising bio-signal for smart drug delivery.

**Figure 1.**
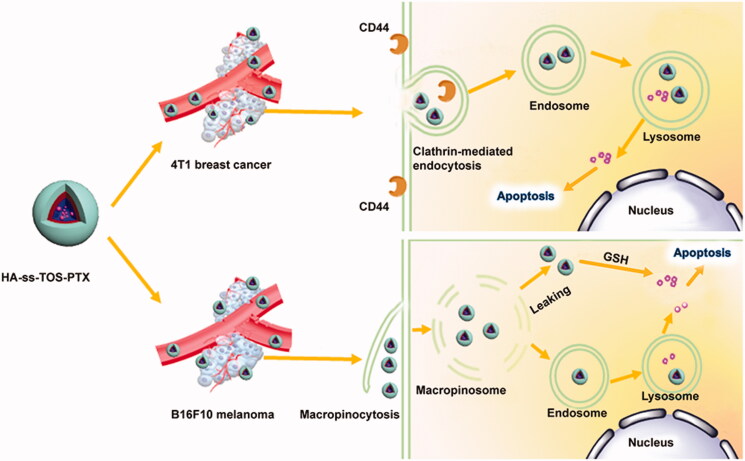
Schematic illustration of different antitumor mechanisms of HA-ss-TOS-PTX against 4T1 cells and B16F10 cell bearing mice, respectively. After being intravenously administrated, HA-ss-TOS micelles rapidly accumulated in 4T1 tumor tissue and B16F10 melanoma cells.

In our previous study (Xia et al., 2018), we constructed GSH-responsive polymeric micelles composed of vitamin E succinate conjugated hyaluronic acid (HA-ss-TOS), which had a desirable average particle size (150 nm) and a high drug loading content (about 37%). The GSH-induced disassembly of HA-ss-TOS-PTX was validated by TEM, drug release behavior as well as a change of particle size in the reducing environment, while the biological evaluation of HA-ss-TOS-PTX in different cancer cells with distinct CD44 expression was not conducted. In the present study, we have chosen A549, B16F10 and 4T1 cells with different CD44 expression to study their cellular internalization mechanism and included antineoplastic studies (Figure 1).

## Results and discussion

### Preparation and characterization of PTX-loaded micelles

The amount of TOS grafting on HA had a large influence on drug loading, drug encapsulation and the particle size of the micelles. Previously, we optimized the preparation process which revealed a preferred degree of substitution (DS, 11%) of TOS in HA-ss-TOS and HA-TOS conjugates and exhibited a desirable particle size (about 150 nm) which were prone to self-assemble into micelles in aqueous media. Moreover, the HA-ss-TOS conjugates and HA-TOS conjugates with 11% DS showed excellent physical compatibility with PTX, and a high drug loading (about 37%) as well as drug encapsulation (about 90%) capability (Xia et al., 2018).

**Figure 2.**
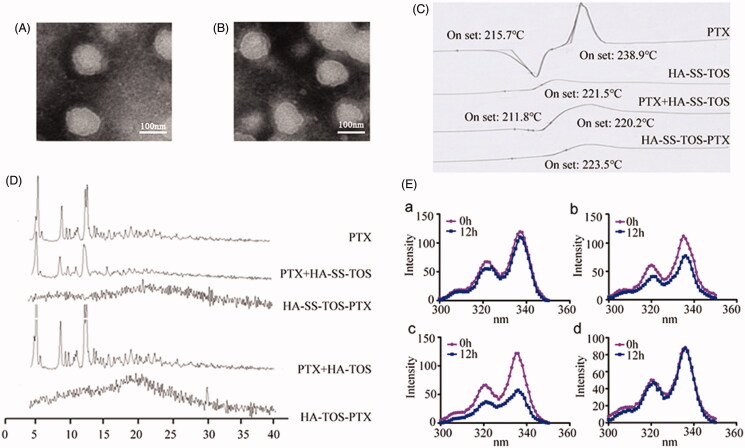
(A) TEM image of the HA-ss-TOS-PTX micelles. (B) TEM image of the HA-TOS-PTX micelles. (C) DSC curves of PTX, HA-ss-TOS, the physical mixture of PTX and HA-ss-TOS, and HA-ss-TOS-PTX. (D) WARD of PTX, the physical mixture of PTX and HA-ss-TOS, HA-ss-TOS-PTX, the physical mixture of PTX and HA -TOS, HA-TOS-PTX. (E) Fluorescence intensity of pyrene in the presence of different concentrations of GSH: (a) HA-ss-TOS without GSH; (b) HA-ss-TOS with 10 mM GSH; (c) HA-ss-TOS with 20 mM GSH; and (d) HA-TOS with 20 mM GSH.

The particle size and morphology of the PTX loaded HA-ss-TOS and HA-TOS micelles were characterized by TEM. Both micelles had a spherical shell-core structure and their particle size was approximate 120 nm (Figure 2(A,B)). The existing state of PTX in the micelles was investigated by DSC. As shown in Figure 2(C), PTX had two T_onset_ values, i.e. 215.7 °C and 238.9 °C, and the T_onset_ of the blank HA-ss-TOS micelles was 221.5 °C. However, after formulation, HA-ss-TOS-PTX exhibited the only T_onset_ at 223.5 °C, indicating the successful encapsulation of PTX into the core of the HA-ss-TOS micelles. XRD analysis showed that PTX had strong peaks at 5.50°, 8.87°, and 12.24° (Figure 2(D)), which also appeared in the XRD spectra of the two pre-mentioned physical mixtures containing PTX. On the contrary, there were no obvious peaks in the XRD spectra of HA-ss-TOS-PTX micelles and HA-TOS-PTX micelles, revealing the complete entrapment of PTX in these two micelles. We found (Figure 2(E)) when pyrene loaded HA-ss-TOS micelles were incubated with 10 mM GSH or 20 mM GSH for 12 h, there was a prominent decrease in fluorescence intensity of pyrene compared to that incubated without the addition GSH. For comparison, pyrene loaded HA-TOS micelles displayed no apparent changes of fluorescence intensity after being incubated with 20 mM GSH. These results demonstrated that the micellar structure of HA-ss-TOS was easily disrupted when exposed to a high concentration of reduction agents.

### Cellular uptake and location of C6-labeled HA-ss-TOS micelles on different oncocytes

As is commonly known, highly expressed CD44 receptors are beneficial to the internalization of HA-covered nanoparticles. Thus, before the cell studies, we tried to validate the CD44 expression level on the surface of different cells, i.e*.,* A549, B16F10 and 4T1 cells. As a result, B16F10 and 4T1cells highly expressed CD44, accounting for approximately 73.2% and 91.5%, respectively, while A549 cells showed extremely lower CD44 expression with a mere 8.5% (supplementary Figure S1). The HA based active targeting nanomedicines have been a research hotspot in the field of cancer therapy duo to the enhanced targeting efficacy and improved antineoplastic activities (Lv et al., 2018; Paidikondala et al., 2019; Phua et al., 2019). 4T1, B16F10 and A549 cells were chosen for this research and we have figured out B16F10 cells were highly expressed CD44 proteins, while A549 cells showed low expression of CD44 proteins, which were exploited for the biological evaluation of a redox-sensitive hyaluronic acid-based nanomedicine

**Figure 3.**
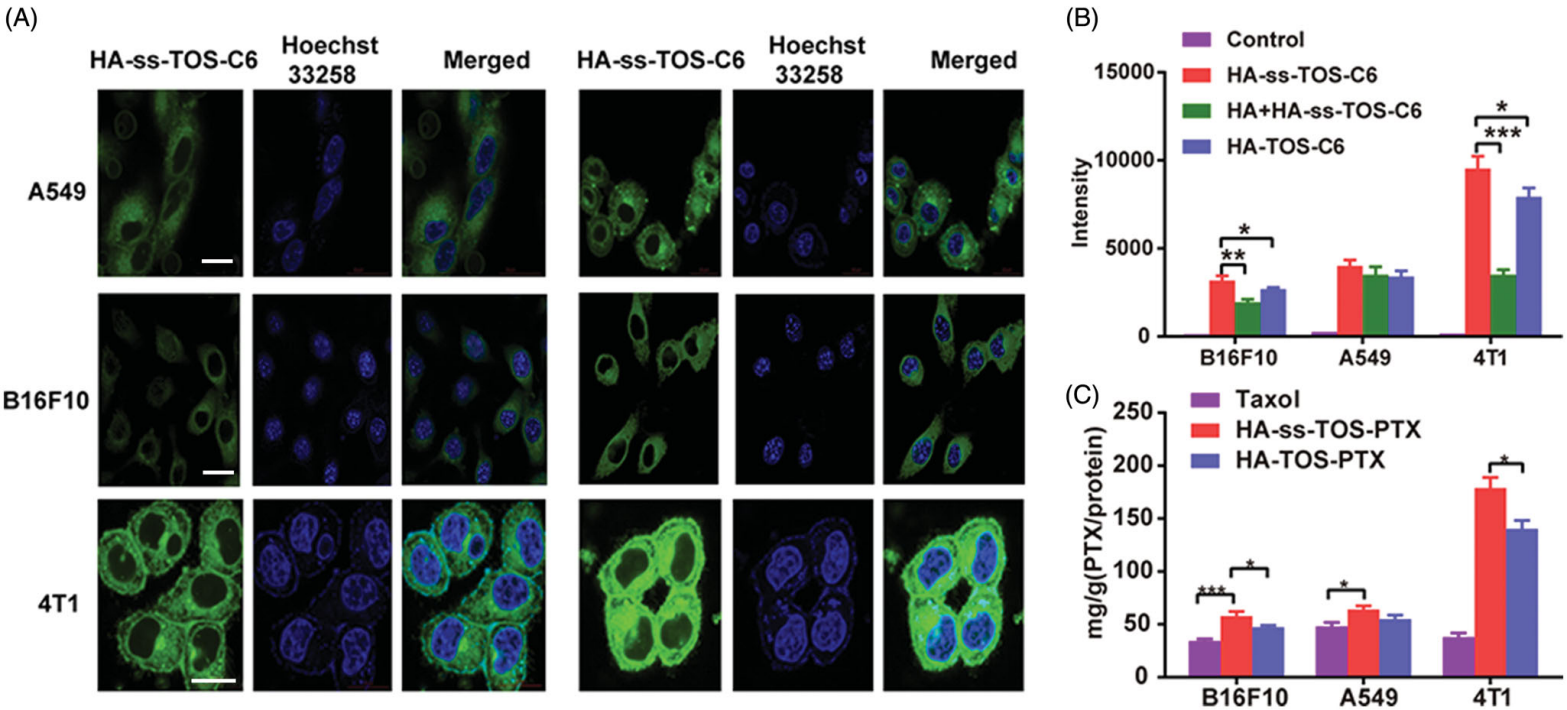
(A) CLSM images of A549, B16F10 and 4T1 cells after 1 h and 4 h in incubation with HA-ss-TOS-C6 micelles. Scale bars are 10 μm. (B) Intracellular uptake of HA-ss-TOS-C6 micelles, free-HA polymer pretreated HA-ss-TOS-C6 micelles and HA-TOS-C6 micelles at 1 h upon incubation with B16F10, A549 and 4T1 cells. (C) Intracellular uptake of HA-ss-TOS-PTX micelles, HA-TOS-PTX micelles and Taxol at 4 h upon incubation with B16F10, A549 and 4T1 cells. *p < .05, **p < .01, ***p < .001.

The CLSM results in Figure 3(A) showed that the HA-ss-TOS-C6 micelles were mainly located in the cytoplasm which was the effective target site of PTX in A549, B16F10 and 4T1 cells. The results achieved by flow cytometry showed that the mean fluorescent intensity of the HA-ss-TOS-C6 micelles in the 4T1 cells was almost triple as that in B16F10 cells and approximately 2.5 times higher than that in A549 cells (Figure 3(B)), which was consistent with the CLSM results. Furthermore, as was shown in Figure 3(B), we found the addition of free HA (10 mg/mL) dramatically decreased (*p* < .001) the fluorescent intensity of HA-ss-TOS-C6 micelles in 4T1 cells and a lower green fluorescence (*p* < .01) was measured after treatment with free HA in B16F10 cells. However, there was no distinct change in fluorescent intensity in the presence of HA in A549 cells. These results implied that free HA could competitively restrain the uptake of HA-ss-TOS-C6 micelles by B16F10 and 4T1 cells. Finally, it was found that cells highly expressing CD44, especially 4T1 cells (*p* < .001), were inclined to take up HA-ss-TOS-PTX more than Taxol (Figure 3(C)).

### Cellular uptake mechanisms of C6 -labelled HA-ss-TOS micelles on different oncocytes

**Figure 4.**
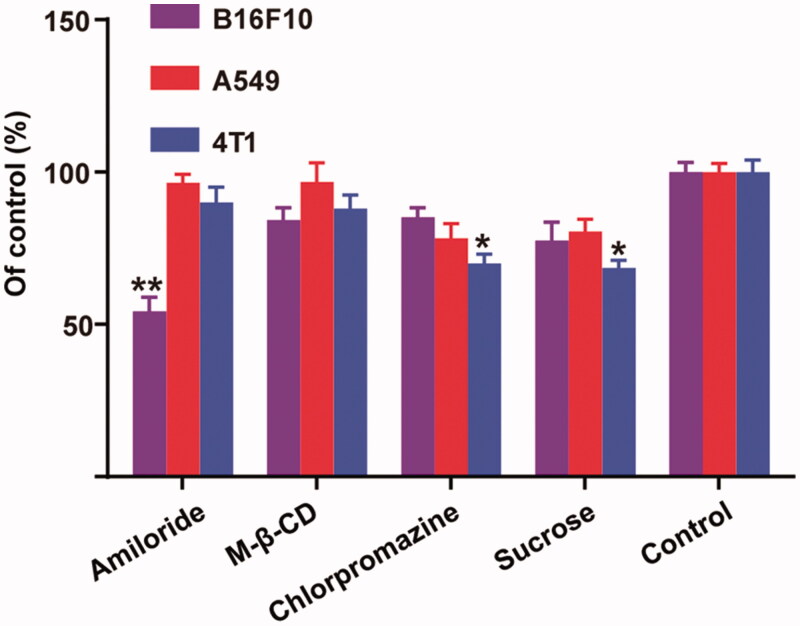
Relative internalization efficiency of HA-ss-TOS-C6 micelles by B16F10, A549 and 4T1cells in the presence of various endocytosis inhibitors. *p < .05 vs. control and **p < .01 vs. control.

As shown in Figure 4, the addition of sucrose and chlorpromazine significantly decreased the uptake of micelles in 4T1 cells (*p* < .05), which implied that a clathrin-mediated internalization should be the major pathway for 4 T-1 cells. To our surprise, amiloride was able to dramatically block the endocytosis of micelles by B16F10, while no distinct changes were observed for 4T1 and A549 cells. Thus, we understood that the micelles were taken up by B16F10 cells mainly via micropinocytosis, which can explain the negligible influence of the addition of free HA on the internalization of micelles into B16F10 cells.

**Figure 5.**
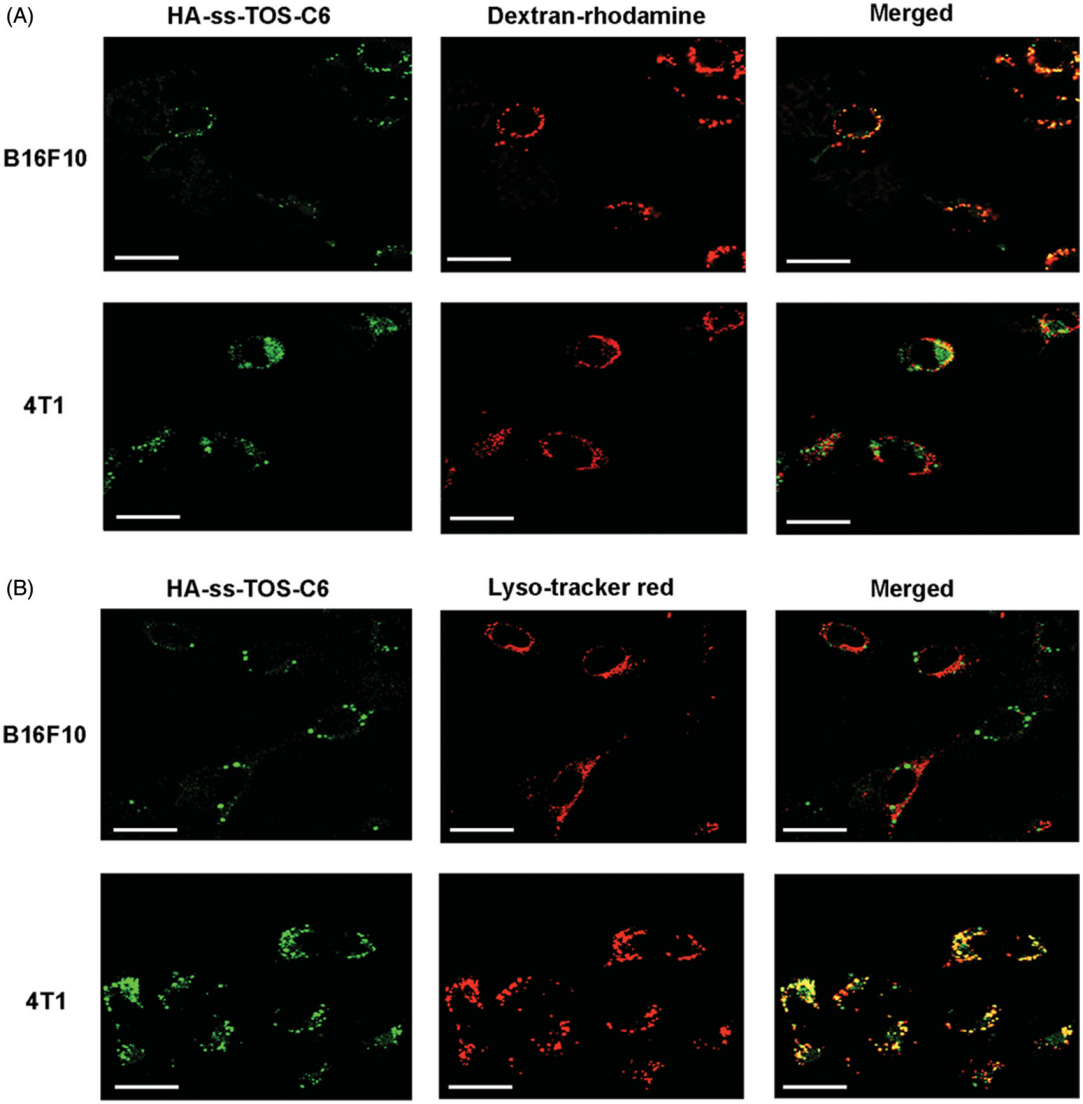
(A) Co-localization of the micelles into macropinosomes of 4T1 and B16F10 cells at 30 min as observed by CLSM. The macropinosomes were stained with Dextran-rhodamine. Scale bars are 20 μm. (B) Co-localization of the micelles into endo/lysosomes of 4T1 and B16F10 cells at 30 min as observed by CLSM. The endo/lysosomes were stained with Lyso-tracker red. Scale bars are 20 μm.

The CLSM images in Figure 5(A) displayed that a strong yellow fluorescence appeared in the B16F10 cells, while only a weak yellow fluorescence was observed in the 4T1 cells. These data further validated the micropinocytosis pathway of B16F10 to translocate the HA-ss-TOS micelles. As shown in Figure 5(B), intensive yellow was observed when the green fluorescence of HA-ss-TOS-C6 was overlaid with the red fluorescence from the Lyso-tracker red in the 4T1 cells, while no obvious yellow was observed in the B16F10 cells. These results indicated that HA-ss-TOS-C6 was endocytosed by 4T1 cells via a clathrin-mediated route.

We found high expression of CD44 is beneficial for the internalization of micelles covered with an HA shell, while 4T1 and B16F10 cells displayed such a big difference in cellular uptake although they both expressed a high level of CD44. To figure it out, we have demonstrated 4T1 cells and exerted different cellular internalization mechanisms. More specifically, HA-ss-TOS-C6 was endocytosed by 4T1 cells via the clathrin-mediated route which enhanced the cell uptake by 4T1 cells via interaction of CD44 with HA while, interestingly, macropinocytosis of HA-ss-TOS-C6 into B16F10 melanoma cells was found, which was identical to the previous report that inducible macropinocytosis of HA in B16F10 melanoma cells (Greyner et al., 2010).

### *In vitro* antineoplastic effects

**Figure 6.**
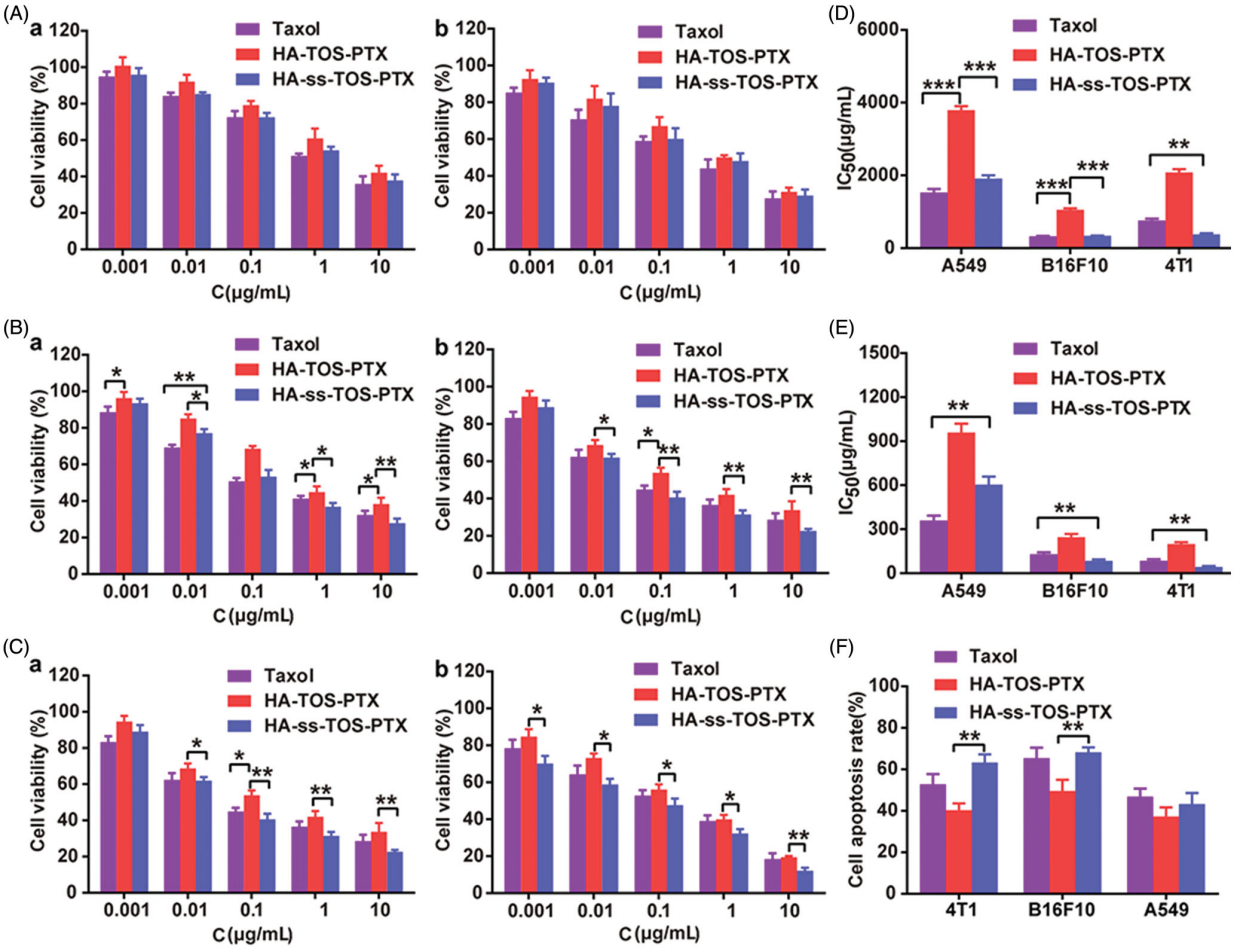
Anti-proliferative activity of (A) A549 cells, (B) B16F10 cells, and (C) 4T1 cells for (a) 24 h and (b) 48 h. IC_50_ values calculated from the cytotoxicity of Taxol, HA-TOS-PTX and HA-ss-TOS-PTX micelles against A549, B16F10 and 4T1cells after (D) 24 h and (E) 48 h. (F) Apoptosis of B16F10, A549 and 4T1 cells observed by CLSM after treatment with Taxol, HA-TOS-PTX and HA-ss-TOS-PTX at a PTX concentration of 1 μg/mL for 24 h. *p < .05, **p < .01, ***p < .001.

The anti-proliferative effects of PTX-loaded micelles against cancer cells were evaluated via the MTT method. Different from the A549 cells and B16F10 cells, 4T1 cells were more sensitive to HA-ss-TOS-PTX micelles rather than Taxol and HA-TOS-PTX micelles even at a low concentration, i.e. 0.001 μg/mL (Figure 6(A–C)). The blank HA-ss-TOS micelles exerted a synergistic antineoplastic effect with PTX against B16F10, A549, and 4T1 cells (Supplementary Figure S2). In comparison, blank HA-TOS micelles exhibited lower antineoplastic activities. Furthermore, both of the blank HA-ss-TOS micelles and HA-TOS micelles showed no significant cytotoxicity against L-02 cells, suggesting that the redox-sensitive nanocarrier exerted synergistic anti-cancer effects with PTX and were nontoxic to normal cells (Supplementary Figure S2).

The IC_50_ values calculated from the MTT results in Figure 6(D,E) revealed that, compared to inert HA-TOS micelles, redox-responsive HA-ss-TOS-PTX micelles exhibited 2 times lower cell viability in A549 cells, 3.1 times lower in B16F10 cells, and 5.5 times lower in 4T1cells, after 24 h of incubation. Apparently, HA-ss-TOS-PTX micelles showed the best antitumor effect against 4T1 cells (Figure 6(F)). When compared to A549 cells, HA-ss-TOS-PTX micelles exhibited a stronger inhibition effect on B16F10 cells. The apoptosis experiment demonstrated that the HA-TOS-PTX group always displayed the lowest cell apoptosis among all groups, which was primarily due to the intracellular slow drug release. Besides, apoptotic bodies in B16F10 and 4T1 cells could be observed with the obvious karyopyknosis after treatment with PTX formulations (Supplementary Figure S3).

*In vitro* cytotoxicity revealed HA-ss-TOS-PTX micelles showed the best antitumor effect against 4T1 cells. 4T1 cells overexpressed CD44 and internalized HA-covered micelles via endocytosis. Thereafter, when exposed to a high concentration of GSH in endosomes/lysosomes, HA-ss-TOS-PTX micelles could be easily disassembled and release PTX. For B16F10 cells compared to A549 cells, HA-ss-TOS-PTX micelles exhibited a stronger inhibition effect on the former. Taking the macropinocytosis pathway and porous membrane structure of macropinosomes into account (Yuan et al., 2012; Mo et al., 2013), HA-ss-TOS-PTX micelles could simply diffuse from the vesicle into the cytoplasm after being taken up by B16F10 cells. Moreover, the cytoplasm is the site of GSH synthesis and has a higher level of GSH compared to other subcellular organelle (Cheng et al., 2015), thus, inducing the disassembly of HA-ss-TOS-PTX micelles resulting in drug release and toxicity to B16F10 cells.

### *In vivo* tumor targeting ability and pharmacokinetics of HA-ss-TOS micelles

**Figure 7.**
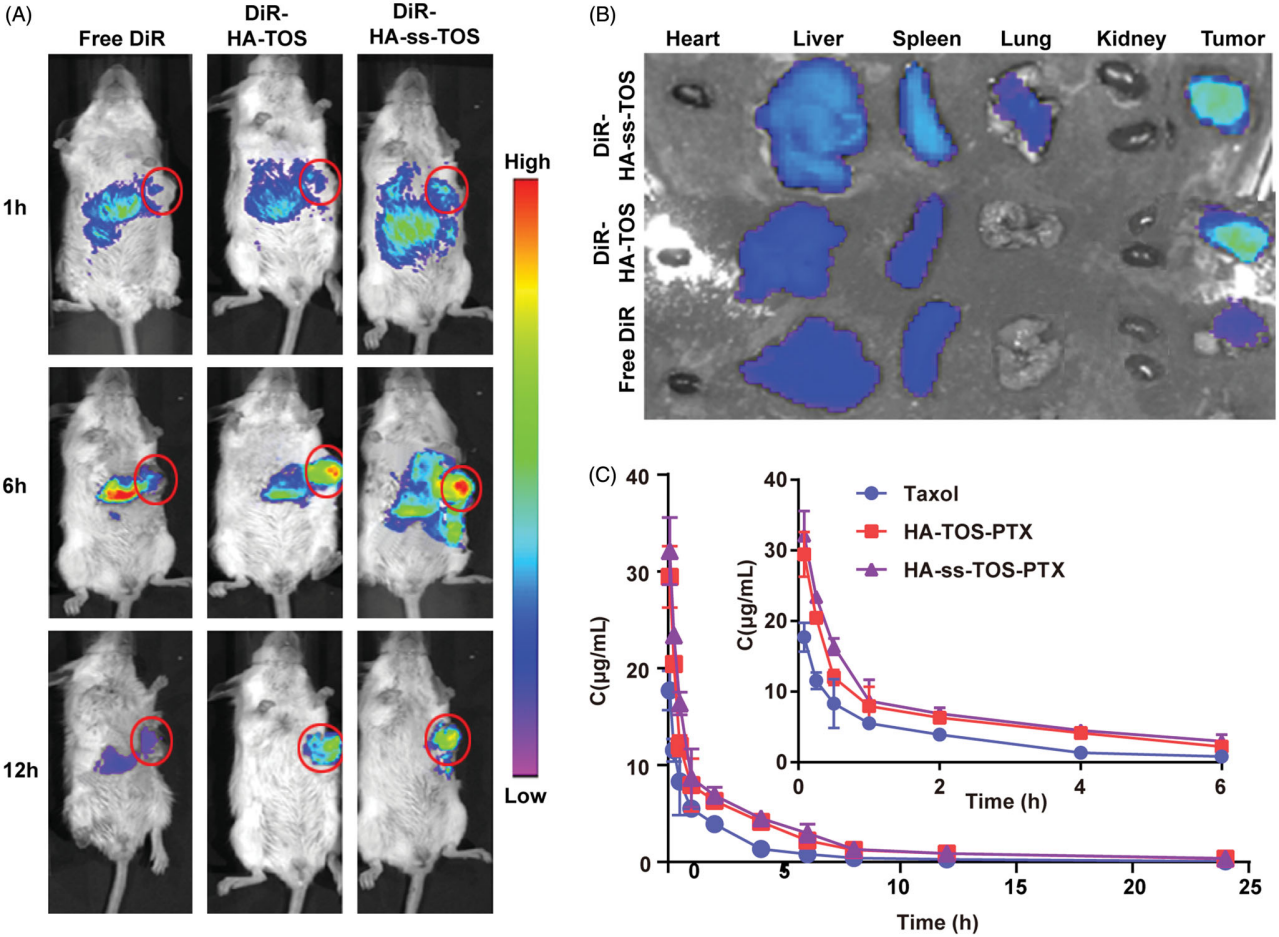
(A) *In vivo* imaging of DiR-loaded formulations in 4T1 tumor-bearing mice. Tumor sites were marked by pink circles. (B) *Ex vivo* imaging of the isolated organs in mice. (C) The change of PTX concentration over a period of time (*n* = 5).

Figure 7(A) revealed that a strong fluorescence was also observed in the tumor site after 6 h post-injection of DiR-HA-TOS and DiR-HA-ss-TOS micelles, while a negligible tumor targeting effect was found for free DiR. Moreover, a much stronger fluorescent signal in tumors at 12 h vs. at 6 h revealed a prolonged circulation time and a tumor-targeting ability of both micelles, which can be also regarded as a powerful proof of great *in vivo* stability for these micelles. Besides, the micelles had a prolonged tumor retention period for more than 24 h, reflecting a potential long-term action of the nano-materials for tumor therapy. On the contrary, free DiR showed negligible tumor accumulation and quick clearance from the body. The ex vivo fluorescent images taken by IV-IS demonstrated a consistent result with the *in vivo* data (Figure 7(B)).

The pharmacokinetic curves (Figure 7(C)) of HA-ss-TOS-PTX and HA-TOS-PTX micelles presented a slower downtrend when compared to Taxol, indicating that the PTX-loaded micelles decreased blood clearance and had a prolonged blood circulation time. The pharmacokinetic parameters calculated by the PK Solver are shown in Supplementary Table S1. Taxol showed the lowest Mean Retention Time (MRT) (*p* < .05) when compared to HA-ss-TOS-PTX and HA-TOS-PTX micelles. The AUC_0-t_ of Taxol was 1.9 times and 1.7 times lower than HA-ss-TOS-PTX micelles and HA-TOS-PTX micelles, respectively. HA based micelles performed slower drug clearing rate than Taxol (*p* < .01).

The excellent *in vivo* tumor targeting ability and improved pharmaceutics of nanomedicine were helpful to facilitate the efficacy and control the toxicity (Sun et al., 2017). HA-ss-TOS-PTX exhibited a superior tumor targeting ability and dramatically prolonged the circulation time of PTX. It was mainly due to the polysaccharide with abundant carboxyl side chains (Zhong et al., 2019). Hydrophilic HA was used to form the shell of the micelles and make them negative. Thus, the negatively charged feature would lower the opportunity of micelles to interact with the anionic plasma protein and be trapped by the reticuloendothelial system (RES) in the liver.

### Evaluation of *in vivo* anti-tumor activities and systemic toxicity

**Figure 8.**
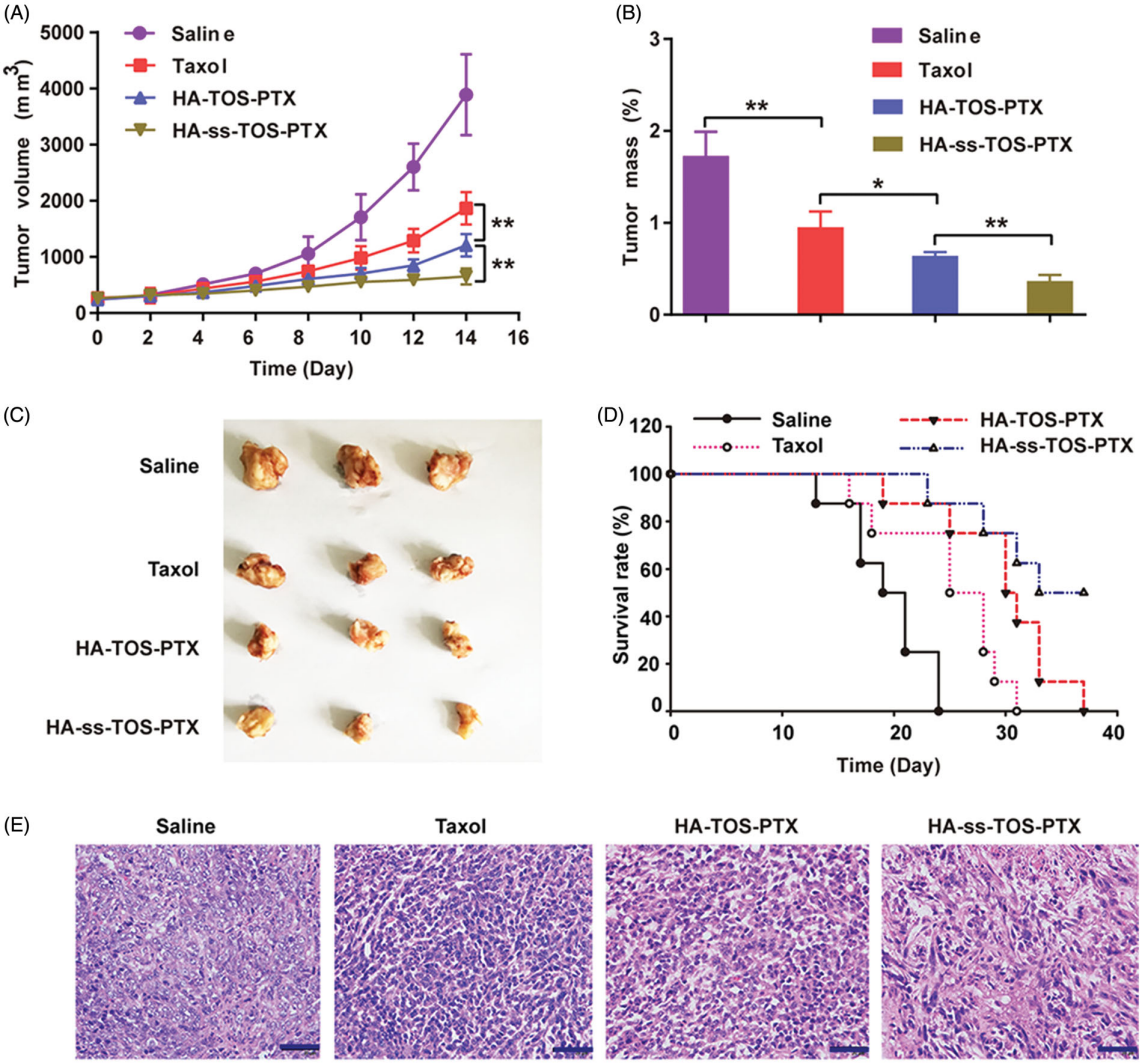
(A) The growth of tumors after being treated with saline, Taxol, HA-TOS-PTX and HA-ss-TOS-PTX (*n* = 11). (B) The weight of isolated tumor tissues from mice treated with saline, Taxol, HA-TOS-PTX, and HA-ss-TOS-PTX after 2 weeks (*n* = 3). (C) Images of isolated tumor tissues from mice treated with saline, Taxol, HA-TOS-PTX, and HA-ss-TOS-PTX after two weeks. (D) The survival rate of mice treated with saline, Taxol, HA-TOS-PTX, and HA-ss-TOS-PTX (*n* = 8). (E) The HE staining of isolated tumor tissues treated with saline, Taxol, HA-TOS-PTX and HA-ss-TOS-PTX after 2 weeks. Scale bars are 100 μm. *p < .05, **p < .01.

As previously mentioned, 4T1 cells overexpressed CD44, which was closely related to the internalization and cytotoxicity of the HA-covered micelles. Thus, 4T1 cells were supposed to be the most suitable cell line to establish the tumor-bearing animal model and investigate the *in vivo* antitumor performance of our preparations. The tumor growth rate significantly slowed down after treatment with the PTX-loaded formulation and the HA-ss-TOS-PTX micelles showed the strongest tumor inhibition efficacy among all groups (Figure 8(A)). Mice receiving HA-ss-TOS-PTX micelles had the smallest tumors after a 2-week treatment (Figure 8(B,C)). In addition, the intravenously administered micelles raised the survival rate and markedly prolonged the mean survival time of 4T1 cell-bearing mice (Figure 8(D)). After sectioning and HE staining of the harvested tumor tissues, it was obvious that the HA-ss-TOS-PTX micelles successfully induced cancer cell apoptosis at a significant measure. In addition, HA-ss-TOS-PTX micelles could dramatically extend the survival time of B16F10 melanoma bearing mice (Supplementary Figure S4). Meanwhile, no significant weight loss was observed after injection with micelles and saline other than Taxol formulation (Supplementary Figure S5). Moreover, HA-ss-TOS-PTX also exhibited inappreciable stimulation (Supplementary Figure S6) and the maximum tolerable dose (Supplementary Table S2).

4T1 breast cancer and B16F10 melanoma models were exploited for evaluation of anti-tumor efficacy. The therapeutic HA-ss-TOS-PTX effectively halted the growth of aggressive 4T1 breast tumor in comparison with HA-TOS-PTX micelles (*p* < .01) and Taxol (*p* < .001). In addition, the administrated intravenously HA-ss-TOS-PTX raised the survival rate and markedly prolonged the survival time of 4T1 cell-bearing mice and B16F10 cell-bearing mice, respectively. Finally, HA-ss-TOS-PTX exhibited inappreciable toxicity at the treatment dose *in vivo* demonstrating HA-ss-TOS-PTX was biocompatible and nontoxic nanocarrier for PTX delivery.

## Conclusions

In this study, the biological evaluation of a redox-sensitive hyaluronic acid-based nanomedicine was intensively investigated on cancer cells with diverse expressed CD44 proteins. We found that despite different cellular internalization mechanisms, hyaluronic acid-based nanomedicine showed strong antineoplastic effects on 4T1 and B16F10 cells due to redox responsiveness. The enhanced therapeutic effect of HA-ss-TOS-PTX on breast cancer and melanoma by comparison to HA-TOS-PTX and Taxol supports the applicability of redox responsiveness and different cellular internalization mechanisms resulting in a similar antitumor efficacy. Thus, it is strongly suggested to develop relationships between the physicochemical characteristics of nanomedicines and the biological characteristics of diseases to achieve desirable therapeutic outcomes in the clinic.

## Supplementary Material

Supplemental Material
