# Supplementary material for: Enhanced cytotoxicity of a redox-sensitive hyaluronic acid-based nanomedicine toward different oncocytes via various internalization mechanisms
Source: Drug Deliv. 2020 Jan 2;27(1):128–36. doi: 10.1080/10717544.2019.1709919 (PMC6968516; doi:10.1080/10717544.2019.1709919)
Supplement: Supplemental Material [file IDRD_A_1709919_SM2664.docx]

**Enhanced cytotoxicity of a redox-sensitive hyaluronic acid-based nanomedicine towards different oncocytes via various internalization mechanisms**

Yunai Du, Sheng Wang, Tianhao Zhang, Dongsheng He, Jiasheng Tu*, Yan Shen*

Center for Research Development and Evaluation of Pharmaceutical Excipients and Generic Drugs, Department of Pharmaceutics, School of Pharmacy, China Pharmaceutical University, 24 Tong Jia Xiang, Nanjing 210009, China

*Corresponding author. Tel.: +86-25-83271305. E-mail: jiashengtu@aliyun.com; shenyan19820801@126.com.

**Materials and methods**

**Materials**

Coumarin 6 (C6) and Hoechst 33342 were purchased from Sigma-Aldrich (Saint Louis, Missouri, United States). Paclitaxel was purchased from hodoyew Co. LLC. (Nanjing, China). RPMI-1640 culture medium and 0.25% tyrisin were purchased from HyClone (Los Angeles, USA). Fetal bovine serum (FBS) was purchased from CLARK Bioscience (Virginia, USA). RIPA Lysis Buffer and Annexin V-FITC Apoptosis Detection Kit were purchased from NanJing KeyGen Biotech Co. Ltd (Nanjing, China). BCA Protein Assay Kit and Lyso-Tracker Red were purchased from Beyotime Biotechnology (Shanghai, China). HA-ss-TOS conjugates and insensitive HA-TOS conjugates were synthesized as described in our previous report. Briefly, HA-ss-TOS was synthesized by the bridge of disulfide-containing cystamine, grafted with TOS to form amphiphilic polymers. While HA-TOS conjugates were obtained by using adipic dihydrazide as a linker. All the experimental protocols were approved by the Institutional Animal Ethics Committee, and all care and handling of animals were carried out in accordance with the requirements by the Institutional Animal Ethics Committee.

**Preparation and characterization of** **HA-ss-TOS-PTX and HA-TOS-PTX micelles**

For HA-ss-TOS-PTX and HA-TOS-PTX, the micelles were prepared as described previously. In brief, 6 mg of PTX in ethanol was added drop-wise into a 3 mg/mL HA-ss-TOS solution with gentle agitation. The mixture solution was sonicated for 30 min at 250 W (3 s on, 2 s off) at 4°C by an ultrasonicator (probe type). The solution was dialyzed overnight before eliminating the free drugs by centrifugation. The final micellar solution was filtrated through a 0.45 μm porous membrane and was lyophilized. The morphology and particle sizes of the polymeric micelles were observed by transmission electron microscopy (TEM, H7650, Hitachi, Japan). The PTX amounts in the micelles were determined by HPLC (LC-200, Shimadzu, Japan). The entrapment efficiency (EE) and drug-loading (DL) of PTX loaded micelles were calculated by the following equations:

$EE=\frac{Amount of PTX in micelles}{Amount of PTX fed initially}\times100\%$,

$$DL=\frac{Amount of PTX in micelles}{Amount of PTX in micelles+Amount of polymers in micelles}$$

The structural integrity of HA-ss-TOS micelles in the presence of different GSH concentrations was investigated by measuring the fluorescence intensity of pyrene. 1 mg of the HA-ss-TOS or HA-TOS polymers were respectively added into a 10 ml volumetric flask with 6×10^-6^ μM pyrene. The polymer solutions were sonicated for 30 min followed by equilibration overnight. The micellar solutions containing different amounts of GSH (0, 10 μm, 10 mM, and 20 mM) were placed in a water bath at 37℃ for 12 h. Fluorescence spectra were measured by a fluorescence spectrometer (RF-530 PC, Shimadzu, Japan).

**Validation of CD44 expression on the surface of different tumor cells**

CD44 expression on B16F10 cells was determined via flow cytometry.^1^ Briefly, B16F10 cells were seeded in 6-well plates and cultured in a CO_2_ incubator. After 24 h, these cells were digested with 0.25% trypsin. B16F10 cells were adjusted to a concentration of 10^6^ per 200 μL in each eppendorf tube prior to the addition of 10 μL anti-CD44-PE solutions. Cells were obtained through centrifugation at 1000 rpm for 5 minutes and washed with PBS three times. 300 μL of PBS were added to obtain the cell suspension. The fluorescence intensity of B16F10 cells was measured by a MACSQuant flow cytometry. The CD44 expression on the membrane of the A549 cells and 4T1 cells were determined following the same routine.

**Cellular uptake and location of C6-labeled HA-ss-TOS micelles in cancer cells**

Coumarin 6 (C6) was chosen as the hydrophobic fluorescent probe of micelles.^2^ C6 loaded HA-ss-TOS micelles (HA-ss-TOS-C6) were prepared *via* the ultrasonic-dialysis method and C6 loaded HA-ss-TOS micelles (HA-ss-TOS-C6) were prepared for controls. B16F10, 4T1 and A549 cells were harvested by trypsinization and diluted to 5×10^5^ per mL. 2 mL of the dilutions containing different cells were seeded into confocal dishes. Cells were incubated for 24 h. The medium was removed and 0.2 μg/mL C6 in HA-ss-TOS-C6 and HA-TOS-C6 micellar solutions were added. 6-well plates were then returned to the cell culture incubator. After 1 h or 4 h of incubation, the overlying culture media containing the micellar solution were thrown away and washed three times with PBS. To each well of the 6-well plate, 1 mL of 10 μg/mL Hoechst 33258 was added. These plates were cultured in an incubator for 30 min and washed 3 times with PBS. The uptake behavior and location of micelles were observed by laser scanning confocal microscopy.

The HA competitive inhibition assays were conducted to confirm the CD44-mediated endocytosis of HA-ss-TOS micelles. Cells were trypsinized and seeded in 6-well plates. After 24 h of incubation, the supernatant culture medium was removed. Micellar solutions containing C6 were diluted to 1 μg/mL and added into these plates. One group was incubated with HA for 2 h before the addition of micellar solutions. After 1 h of incubation, the culture medium containing C6 were removed and the plates were washed 3 times with PBS. Cells were gathered in eppendorf tubes suspended with 300 μL of PBS after trypsinization and centrifugation. Fluorescent intensity of each well was measured through flow cytometry.

In the redox-responsive HA-ss-TOS micellar delivery system, PTX was exploited as a hydrophobic anticancer model drug. The uptake of Taxol, HA-ss-TOS-PTX micelles and HA-ss-TOS-PTX micelles by A549, B16F10 and 4T1 cells expressed with different amounts of CD44 protein were also investigated. Cells were trypsinized and seeded in 6-well plates. After 24h of incubation, the supernatant culture medium was removed. Micellar solutions and Taxol containing 30 μg/mL PTX were added respectively and incubated for 4h. Thereafter, the supernatant solutions were discarded and washed with 4℃ PBS thrice. Then, cells were treated with a RIPA lysis buffer. The total cell protein assay was determined by the BCA kit and the PTX in the lysis buffer was measured by HPLC.

**Cellular internalization mechanisms of HA-ss-TOS micelles in B16F10 and 4T1 cells**

To thoroughly investigate the mechanisms of internalization of micelles in cells, different uptake inhibitors were used including a high concentration of sucrose, 10 μg/mL of chlorpromazine, 7.5 mM CD-cyclodextrin and 5 μg/mL of amiloride. Cells were trypsinized and seeded in 6-well plates. After 24 h of incubation in a cell culture incubator, the supernatant culture medium was removed and different inhibitors were added. These 6-well plates were returned to the incubator. After 30 min of incubation, the supernatant cell medium was cast away and washed 3 times with PBS and subsequently replaced with culture medium containing C6. After 1 h of incubation in a cell culture incubator, the culture medium containing C6 was removed and the plates were washed 3 times with PBS. Cells were gathered in eppendorf tubes with 300 μL of PBS after trypsinization and centrifugation, respectively. Fluorescence intensity of each well was measured through flow cytometry.

The macropinocytosis of HA-ss-TOS-C6 by B16F10 cells was investigated using a laser scanning confocal microscope. Briefly, cells (B16F10 cells, 4T1 cells, and A549 cells) in suspension were harvested by trypsinization and cells were diluted with culture medium at 5×10^5^ per mL and 2 mL of the dilutions were seeded into confocal dishes. Cells were incubated in a cell culture incubator for 24 h. The supernatant culture medium was removed and 0.1 μg/mL C6 in HA-ss-TOS-C6 and HA-TOS-C6 micellar solutions were added. Confocal dishes were returned to the cell culture incubator. The micellar solutions in the cell medium were cast away and confocal dishes were washed 3 times with PBS after 1 h of incubation in a CO_2_ Incubator. Fresh cell medium containing 100 μg/mL of Dextran-Rhodamine (MW 70 kDa) was added to substitute the supernatant PBS before incubation in a CO_2_ incubator for another 30min. The confocal dishes were washed again to remove the remaining Dextran-Rhodamine. The location of HA-ss-TOS-C6 micelles was observed via an LSM 700 laser scanning confocal microscope.

**In vitro cytotoxicity**

MTT cell proliferation assays evaluated the cytotoxicity of the materials against the cultured B16F10 cells, A549 cells, and 4T1 cells. Taxol and HA-TOS-PTX were chosen as control groups to comparatively explore the inhibitory effects of HA-ss-TOS-PTX on the three cancer cells and the MTT assays were conducted according to the following protocol. First, cells were collected and diluted with culture medium at 5×10^4^ per mL. 100 μL of the dilutions were seeded into each well of 96-well plates. Cells were incubated under a culture incubator for 24 h. The supernatant culture medium was removed and replaced with 100 μL of a new medium containing different concentrations of PTX in three formulations. Each group was returned to a cell culture incubator for 24 h and 48 h, respectively. 20 μL of MTT at 5 mg/mL was added into the culture medium containing PTX and control groups. Subsequently, plates were returned to the cell culture incubator. After 4h of incubation, the supernatant culture medium was carefully discarded before 150 μL of DMSO were added. The plates were shaken at low speeds for 10 min to dissolve the purple precipitate. Each plate cover was removed and absorbance in each well, including the blanks, was measured at 490 nm in a microtiter plate reader.

**Apoptosis of TOS tumor cells**

Cells were seeded in 6-well plates and cultured in a CO_2_ incubator under 5%CO_2_ at 37℃, and after 24 h, these cells were digested with 0.25% trypsin. The supernatant culture medium in each well was replaced with 1 μg/mL paclitaxel in Taxol, HA-ss-TOS-PTX and HA-TOS-PTX micellar solutions. There were 6 multiple pores in the negative control group and positive control group. The 6-well plates were returned to a CO_2_ incubator and incubated for 24 h. After that, the supernatant culture medium was thrown away, subsequently digested with trypsin in EDTA and the cells were harvested with 500 μL of the binding buffer after centrifugation at 1000 rpm for 3 min. 5 μL of Annexin V-FITC and the same volume of PI were added into the cell suspension with incubation for 15 mins. Cell apoptosis was measured by means of a flow cytometer.

**In vivo tumor targeting ability**

4T1 tumor cell-bearing mice were used for in vivo tumor targeting ability and Dir was the hydrophobic fluorescent probe encapsulated in polymeric micelles. When the tumor volume was appropriately 800 mm^3^, Dir-loaded HA-ss-TOS micelles were injected via tail vein at a dose of 2.5 mg/kg; free Dir and Dir-loaded HA- TOS micelles were studied as control groups. After 1 h, 6 h and 24 h post-injection, the mice were imaged lightly with isoflurane and biological imaging was quickly conducted on an in vivo Imaging System (IVIS, PerkinElmer). The ex vivo distribution of the fluorescent dye in each organ or tissue including the heart, liver, spleen, lung, kidney, and tumor was also imaged for the analysis of micellar targeting ability.

**In vivo pharmacokinetics of HA-ss-TOS micelles**

The Sprague Dawley (SD) rats weighing appropriately 200 g were used as animal models. The rats were treated with HA-ss-TOS-PTX micelles, HA-TOS-PTX micelles, and Taxol at a dose of 10mg/kg *via* the tail vein. Blood samples, about 0.5 mL in volume, were collected from orbital venous plexus at various times (5 min, 15 min, 30 min, 1 h, 2 h, 4 h, 6 h, 8 h, 12 h, and 24 h). The blood samples were centrifuged immediately at 8000 rpm under 4℃. The supernatant plasma was separated and stored at -20℃ for further use. Before HPLC analysis, the plasma samples were thawed at room temperature. Measure accurately, 150 μL of the plasma sample was added it a 10 mL centrifuge tube containing an internal standard. Subsequently, each centrifuge tube was mixed for 1min to dissolve the internal standard. Tert-butyl methyl ether was added and the centrifuge tube was mixed for another 5 min. 3 mL of the supernatant solution were collected and the solvent was removed by vacuum drying. After that, 150 μL of the mobile phase was added to dissolve the paclitaxel and the internal standard prior to analysis of the HPLC. The pharmacokinetic parameters were calculated according to a non-compartment model using PK Solver 2.0.

**In vivo anti-tumor activities**

Female mice were subcutaneously injected with 0.2 mL of PBS containing 1×10^7^ 4T1 cells near the axilla. When the tumors reached 200 m^3^, the mice were treated with HA-ss-TOS-PTX micelles, HA-TOS-PTX micelles and Taxol at a dose of 5 mg/kg based on PTX every two days. The saline group was used as the negative control. The tumor volumes were measured and calculated by $V={a\times b^{2}}/2$. The mice were sacrificed on day 14, and tumor tissues were collected and weighted. The survival time of the tumor-bearing mice was also investigated. Briefly, mice were treated with HA-ss-TOS-PTX, HA-TOS-PTX, and Taxol at a dose of 5 mg/kg based on PTX every two days, while those receiving saline were regarded as the control. Each group contained eight mice. The death of mice in each group was recorded to analyze survival time.

**Statistical analysis**

Results are given as mean ± S.D. Statistical significance was tested by a two-tailed Student’s t-test. Statistical significance was set at *P < 0.05, **P < 0.01, and ***P < 0.001. The statistical software used was SPSS 19.0.

**References**

1. M. Sato, T. Takagi, R. Okamoto, T. Koshino, H. Goto, J. Ito, K. Yamamoto, H. Makita, T. Hayashi, M. Uesugi and T. Saito, *Modern rheumatology*, 2002, **12**, 18-23.

2. B. Q. Tang, J. L. Zaro, Y. Shen, Q. Chen, Y. L. Yu, P. P. Sun, Y. Q. Wang, W. C. Shen, J. S. Tu and C. M. Sun, *J Control Release*, 2018, **279**, 147-156.

**Supplementary figures**


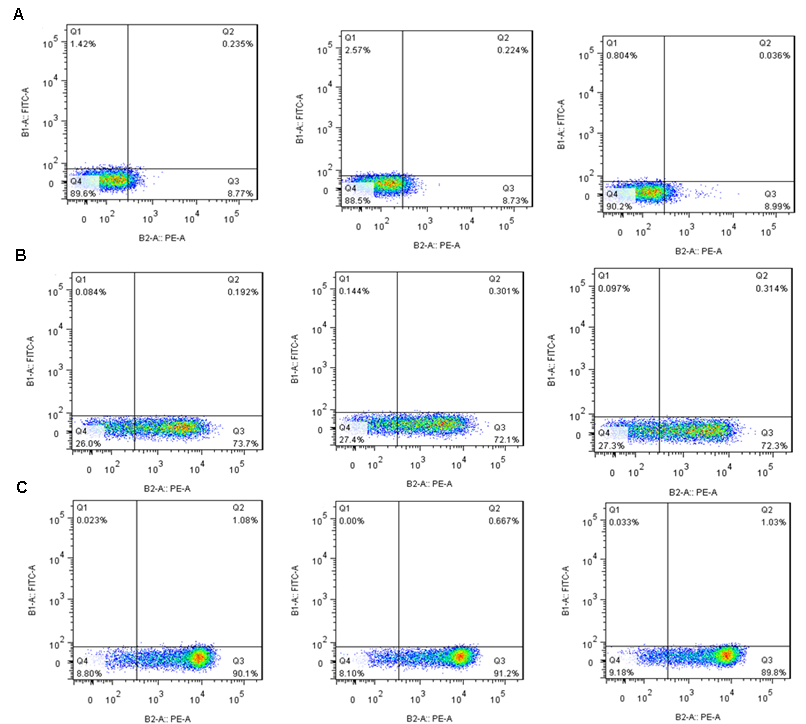


**Figure S1.** Flow cytometric analysis of CD44 expressed on different tumor cells. **(A)** A549 cells; **(B)** B16F10 cells; **(C)** 4T1 cells.


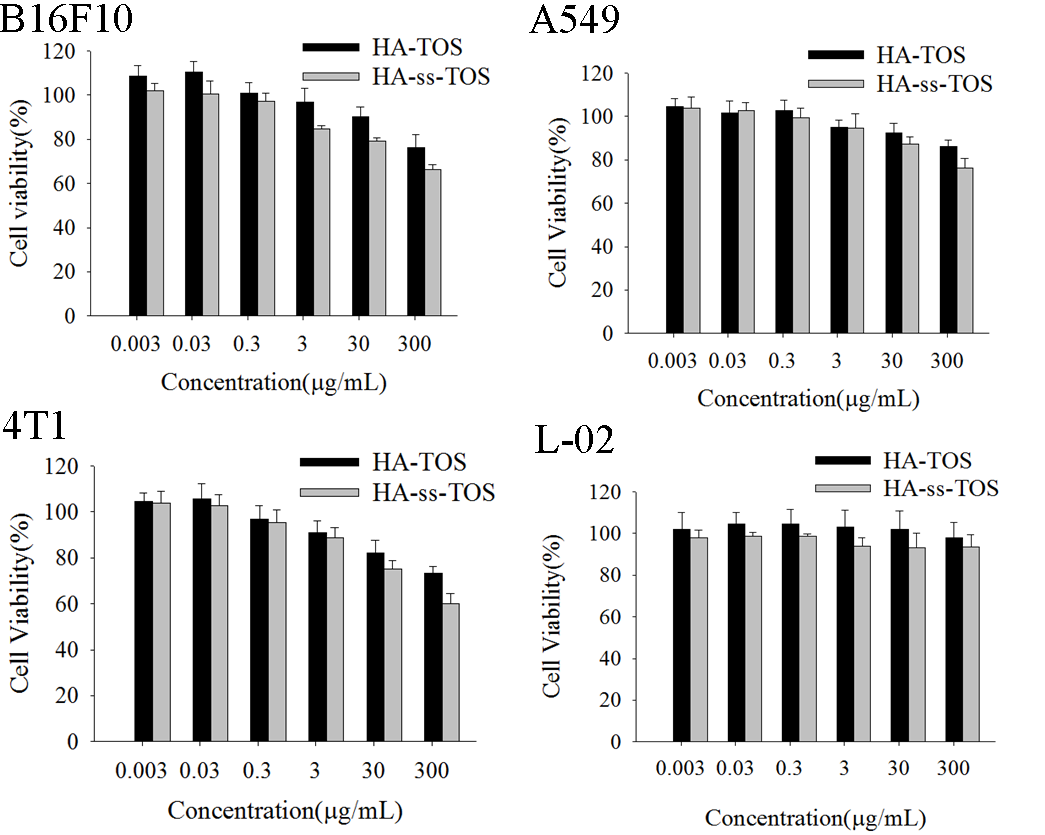


**Figure S2.** Cytotoxicity of blank HA-TOS and HA-ss-TOS micelles towards B16F10, A549, 4T1, and L-02 cells.


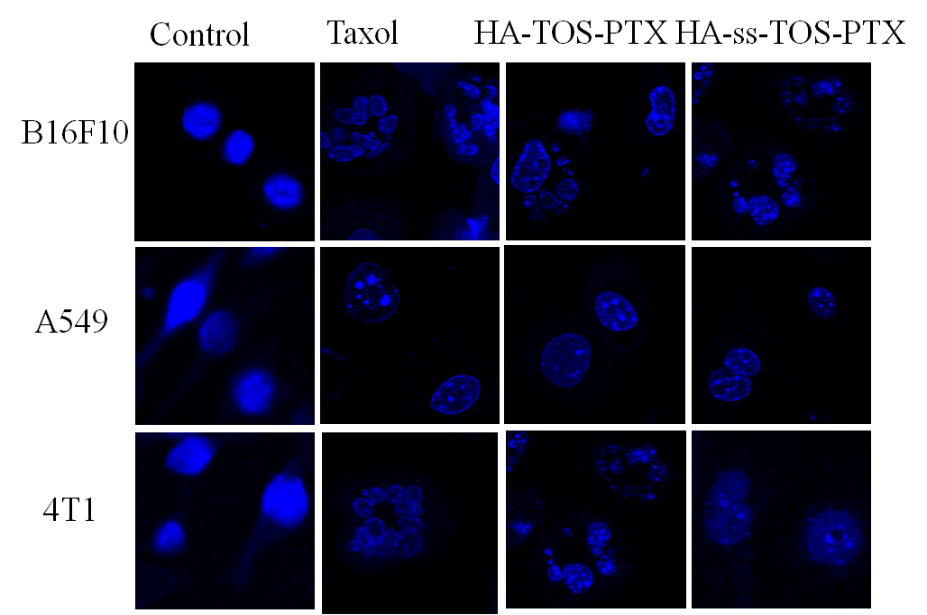


**Figure S3.** Apoptosis of B16F10, A549 and 4T1 cells observed by CLSM after treatment with Taxol, HA-TOS-PTX and HA-ss-TOS-PTX at a PTX concentration of 1 μg/mL for 24h.


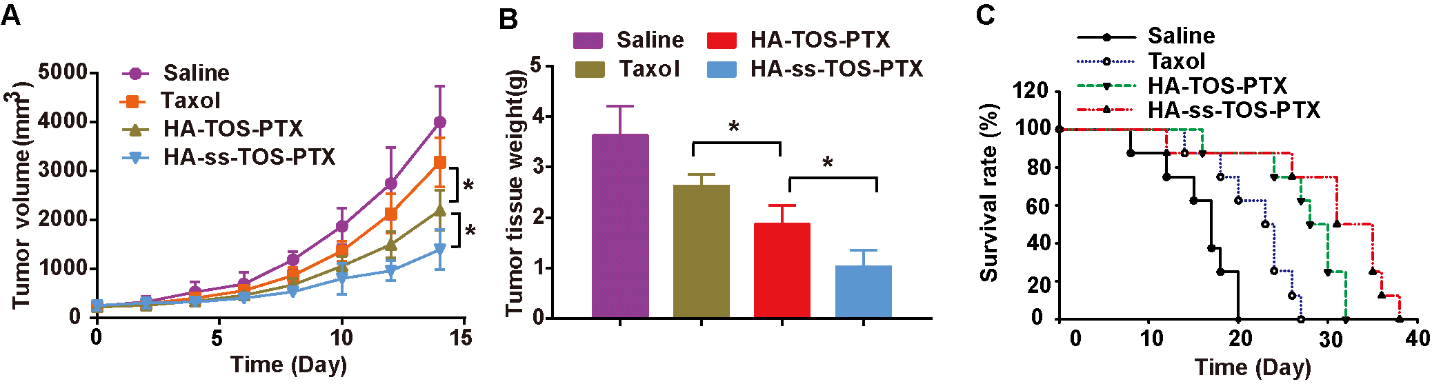


**Figure S4.** (A) The growth of tumors after being treated with saline, Taxol, HA-TOS-PTX and HA-ss-TOS-PTX (n = 11). (B) The weight of isolated tumor tissues from mice treated with saline, Taxol, HA-TOS-PTX, and HA-ss-TOS-PTX after two weeks (n = 3). Survival rate of B16F10 bearing mice treated with saline, Taxol, HA-TOS-PTX and HA-ss-TOS-PTX (n = 8)

.


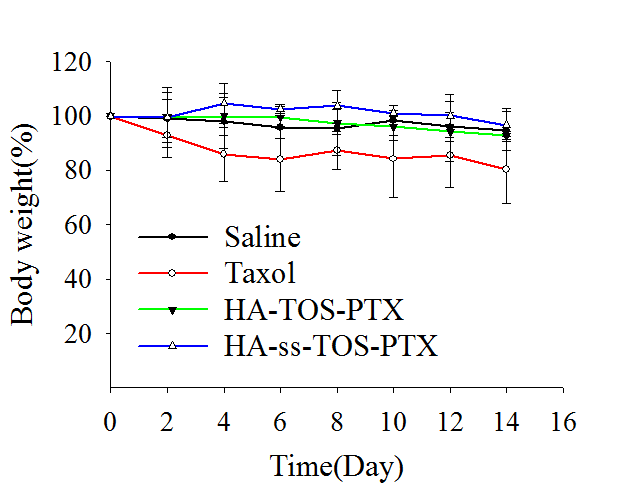


**Figure S5.** Changes of body weight after treatment with saline, Taxol, HA-TOS-PTX and HA-ss-TOS-PTX for two weeks (n = 6).

**
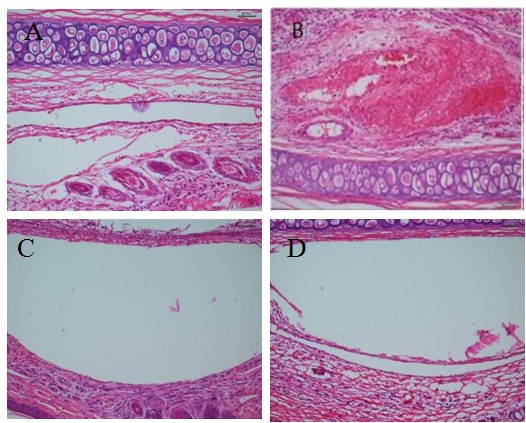
**

**Figure S6.** Vascular stimulation of rabbits after treated with **(A)** Saline, **(B)** Taxol, **(C)** HA-TOS-PTX micelles, and **(D)** HA-ss-TOS-PTX micelles.

**Table S1.** Pharmacokinetic parameters of LND after intravenous injection of different LND formulations at an LND dose of 10 mg/kg in rats. n = 4.

| Parameters | Taxol | HA-ss-TOS-PTX | HA-TOS-PTX |
| --- | --- | --- | --- |
| C_max_ (μg/mL) | 17.7±1.3 | 37.6±5.2** | 32.4±4.6** |
| AUC_0-t_ | 27.0±2.0 | 61.8±6.18** | 55.0±7.7** |
| AUC_0-inf_obs_ | 34.7±1.8 | 66.9±8.5 | 59.4±6.2 |
| MRT（h） | 4.0±1.5 | 7.0±0.9 * | 6.9±0.7* |
| Cl_obs | 29.3±3.7 | 12.3±2.7** | 13.9±3.0** |

*P < 0.05, **P < 0.01 *vs.* Taxol group

**Table S2.** The tolerance dose of Taxol, HA-TOS-PTX, and HA-ss-TOS-PTX on B16F10- bearing mice

| Sample | Dose  (mg/kg) | Death | Reaction after administration | Body weight |
| --- | --- | --- | --- | --- |
| Taxol | 20 | 0/3 | Normality | <10% |
|  | 30 | 0/3 | No strength | <10% |
|  | 40 | 0/3 | No strength | <10% |
|  | 50 | 0/3 | No strength | <10% |
|  | 60 | 3/3 | Death | N.A. |
| HA-TOS-PTX | 75 | 0/3 | Normality | <10% |
|  | 150 | 0/3 | Normality | <10% |
|  | 200 | 0/3 | Normality | >10% |
|  | 250 | 1/3 | Death | >10% |
| HA-ss-TOS-PTX | 75 | 0/3 | Normality | <10% |
|  | 150 | 0/3 | Normality | <10% |
|  | 200 | 0/3 | Normality | <10% |
|  | 250 | 1/3 | Death | >10% |
